# Outcome of early stage Merkel carcinoma treated by exclusive radiation: a study of 53 patients

**DOI:** 10.1186/s13014-021-01815-4

**Published:** 2021-05-14

**Authors:** Manon Dubois, Henry Abi Rached, Alexandre Escande, Frédéric Dezoteux, Franck Darloy, Anaïs Jouin, Maeva Kyheng, Julien Labreuche, Véronique Dziwniel, Xavier Mirabel, Laurent Mortier

**Affiliations:** 1 CHU Lille, Service de Dermatologie, 59000 Lille, France; 2 Univ. Lille, Inserm, CHU Lille, U1189 - ONCO-THAI - Assisted Laser Therapy and Immunotherapy for Oncology, 59000 Lille, France; 3 H. Warembourg, School of Medicine, University of Lille, Lille, France; 4 CARADERM Network, Lille, France; 5 University Department of Radiation Oncology, Oscar Lambret Comprehensive Cancer Center, Lille, France; 6 CRIStAL Laboratory, UMR 9189, University of Lille, Villeneuve d’Ascq, France; 7 Radiotherapy Center, Centre Léonard de Vinci, Dechy, France; 8 Radiotherapy Center, Centre de Cancérologie Les Dentellières, Valenciennes, France; 9 Department of Biostatistics, CHU Lille, 59000 Lille, France; 10 Univ. Lille, CHU Lille, ULR 2694 - METRICS: Evaluation Des Technologies de Santé Et Des Pratiques Médicales, 59000 Lille, France; 11 Languages Department, Centrale Lille Institut, Villeneuve d’Ascq, France

**Keywords:** Exclusive radiation, Merkel cell carcinoma, Skin cancer

## Abstract

**Purpose:**

Early stage Merkel cell carcinoma (MCC) is a rare and aggressive primary skin cancer. The standard of care for MCC is broad excision and adjuvant external beam radiation therapy (EBRT). However, for some patients, anesthesia is contraindicated, while others run the risk of serious aesthetic sequelae. In such cases, exclusive radiotherapy is an interesting alternative to surgery. Though limited data is available, this study evaluates exclusive radiotherapy for MCC, using data from the largest retrospective study to date.

**Methods:**

All patients who were followed in our center between 1989 and 2019 for histologically proven early stage MCC were included in the study. They were treated either by surgery with a 2-cm clear margin followed by adjuvant radiotherapy (RT) or by exclusive RT. Survival rates with adjuvant and exclusive EBRT were analyzed using Cox model and Fine and Gray model depending on the type of survival. *p* value < 0.05 was considered significant.

**Results:**

Eighty-four patients treated for MCC were included. Fifty-three of them (63.1%) were treated by exclusive RT, and 31 (36.9%) had surgical excision followed by adjuvant RT. Local relapse rate was 13.7% (95% CI 8.0–43.7) in the RT monotherapy group (group A) and 25.8% (95% CI 10.3–56.2) in the surgery + RT group (group B) (*p* = *0.42*)*.* No statistical difference was found for nodal relapse (*p* = *0.81*)*,* metastatic relapse (*p* = *0.10*)*,* disease free survival (*p* = *0.83*) or overall survival (*p* = *0.98*).

**Conclusion:**

Our study suggests that exclusive radiotherapy for early Merkel cell carcinoma leads to a similar oncological outcome as combined treatment, with fewer aesthetic sequelae. The approach is interesting for elderly patients with comorbidities or patients for whom surgery would cause significant functional or aesthetic sequelae.

**Supplementary Information:**

The online version contains supplementary material available at 10.1186/s13014-021-01815-4.

## Introduction

Merkel cell carcinoma (MCC) is a rare but aggressive skin cancer. Its overall incidence has increased over the past 20 years and varies between 0.1 and 1.6/100,000 [1]. It frequently affects the elderly, with a mean age of 74.9 years [1] and immunosuppressed patients [2–4]. The primary tumor is frequently located on sun-damaged skin, especially in the head and neck region.

It is an aggressive tumor, with 5-year survival rate of 64% for patients with localized tumors, 39% for patients with tumors with lymph nodes involvement and 18% at the metastatic stage [5]. In addition, patients face an estimated 35% risk of local recurrence, a 40% risk of lymph node recurrence and a 20% risk of metastatic recurrence after surgery [6].

At localized stage, wide surgical excision with a 2–3 cm margin followed by radiotherapy (RT) (doses ranging from 50 to 66 Gy) is the recommended treatment [7, 8]. Post-surgical functional sequelae could majorly alter the quality of life in the elderly population, making exclusive RT an interesting option in this population. Some authors suggest that margins of 1–2 cm may be sufficient, when using Mohs’ micrographic surgery which includes histologic examination of tissue edges and aim to spare as much healthy tissue as possible [8]. In patients with negative clinical lymph nodes, it is recommended to perform a sentinel lymph node biopsy (SLNB) and, if the SLNB is positive, a lymph node resection and RT. At the metastatic stage, chemotherapy or immunotherapy is proposed.

In daily clinical practice, surgical techniques may be limited either by aesthetic and major functional sequelae or by anesthesia contraindication. Exclusive radiotherapy may be an effective alternative, as MCC is known to be a radiosensitive tumor [9]. Indeed, several authors have observed that a treatment of MCC patients by exclusive radiotherapy leads to good outcomes [10–13].

The aim of this study was to compare exclusive radiotherapy to the gold standard treatment (surgery followed by radiotherapy) in localized MCC.

## Methods

### Population and treatment

We reviewed the medical records of patients referred to our department of dermatology and treated with curative intent for MCC. Patients’ data were collected in computerized and anonymized medical files using a unique identifier for each patient, then stored in a secured file for statistical analysis. This included patients with localized MCC, who presented a solitary tumor. Lymph node involvement was assessed by imaging (either by ultrasound or CT scan) and/or by SLNB. Patients with a positive SLNB or lymph node metastasis were excluded. Patients were divided into two groups: group A for patients with a unique, non-resected MCC at the time of RT and group B for patients who had surgery with a 2 cm margin followed by adjuvant RT. Patients treated by surgery with a margin smaller than 2 cm or by surgery without adjuvant RT were excluded. The RT technique varied based on according to the treatment center, the time of treatment and the patient (2D, 3D, intensity-modulated radiotherapy, cobalt or photon radiotherapy).

Our primary objective was to compare the local relapse rate in the two groups. We defined the local relapse as a confirmed MCC relapse in the irradiated area.. Our secondary objectives were to compare the nodal relapse, metastatic relapse, disease-free survival (DFS) and overall survival (OS) rates in the two groups.

### Statistical analysis

Data are expressed as numbers (percentages) for categorical variables, and median and interquartile ranges (IQR) for quantitative variables. We estimated the cumulative incidence of specific recurrence events (local, nodal and metastatic) and specific mortality by using the approach of Kalbfleisch and Prentice [14], treating non-specific death (according study outcome) as competing risk.

We investigated the associations between the type of surgery (i.e., RT monotherapy group and surgery + RT group) and the occurrence of specific recurrences in univariable and multivariable Fine-Gray models adjusted on pre-specified parameters: age, size, radiation dose on the lesion, radiation dose on the draining lymph nodes and localization. All these factors are known as prognostic factors for MCC in the literature.

The rates of overall survival (OS) and disease-free survival (DFS defined as the time to any recurrence of death) were estimated using the Kaplan–Meier method. The impact of the type of surgery was analyzed using the univariable and multivariable Cox model regression with similar prespecified adjustments. The proportional hazard assumption was checked by examining the Schoenfeld residuals [15]. The same pre-specified adjustments as above were applied.

Confidence intervals of 95% using loglog methods (CI95%) were used. All statistical tests were done at the two-tailed α level of 0·05. Data were analyzed using SAS version 9.4 [SAS Institute Inc., Cary, NC 27513, USA].

### Ethics

According to the standard procedure and the Jardé Law (March 2012) on the publication of retrospective data, all the patients of the study expressed their non-opposition to the use of their anonymized medical data. The study has been declared to and accepted by the CNIL [Commission Nationale Informatique et Libertés, the organization in charge of the ethical use of data collected for scientific purposes in France (DEC19-373)].

## Results

### Patients characteristics

**Fig. 1:**
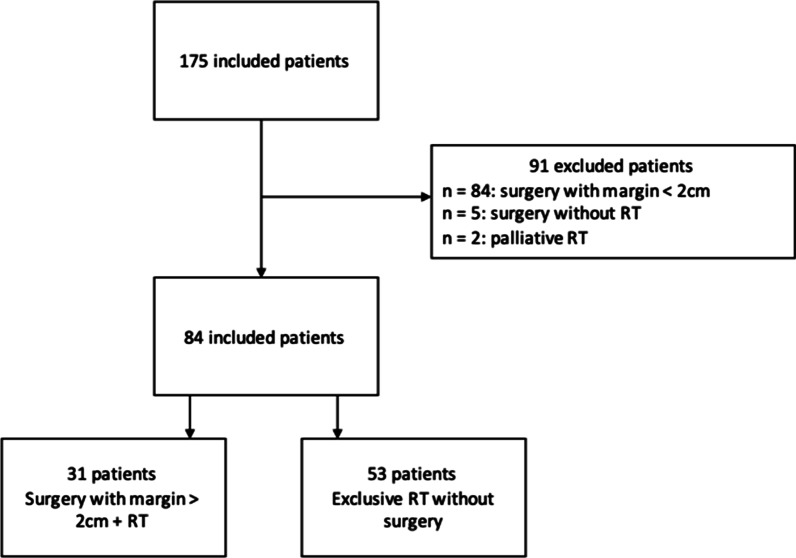
Study flow diagram

Between 1989 and 2019, 175 patients with stage I–II MCC were treated, then underwent clinical and imaging follow-up (lymph node imaging and/or body CTscann) once every 3 months for 3 years then once every 6 months for 2 years. Patients who did not relapse were subsequently followed up with a clinical examination once a year by their dermatologist. Eighty-four patients (48.0% of the 175 patients) were included in the study: Fifty-three (63.1%) in group A and 31 (36.9%) in group B. Of the 91 patients (52.0%) excluded, 84 (92.3% of the 91 patients excluded) were excluded because they had undergone surgery with margins smaller than 2 cm, two (2.1%) were excluded because of palliative or hemostatic RT, and five (5.5%) were excluded because they had undergone surgery without RT. The study flow diagram is shown in Fig. 1.

The median tumor diameter was 2.0 cm [(IQR 1.2–4.0); ranging from 0.4 to 9 cm] in group A and 2.5 cm [(IQR 1.8–4.0); ranging from 1.2 to 8 cm] in group B. The primary tumor was located in the head and neck region for 43 patients in group A (81.1%) and only eight patients in group B (23.3%). Eight patients (15.0%) in group A had hematological diseases (5 chronic lymphocytic leukemias and three lymphomas), compared to three patients (9.7%) in group B (One chronic lymphocytic leukemia and one lymphoma). The patients in group A were ineligible for surgery either because of the location and/or size of the tumor (32 out of 41 patients, or 78.0%, missing data for 12 patients) making surgery too decaying or because comorbidities or bad general conditions contraindicating anesthesia (8/41 patients, or 19.5%). One patient refused surgery. The patients’ baseline characteristics are presented in Table 1.

**Table 1:** Baseline demographics and clinical characteristics of patients n: number; y: years; Gy: Gray TNM (tumor; nodes; metastatic) staging of Merkel Carcinoma in AJCC 8th Edition 2016

	Overall patients	Exclusive RT (group A)	Surgery + RT (group B)	*p* value
	n = 84	n = 53	n = 31	
Sex, n (%)				
Male	26 (31.0)	13 (24.5)	13 (41.9)	0.096
Female	58 (69.0)	40 (75.5)	18 (58.1)	
Median age, y (IQR)	79.0 (72.0–85.0)	82.0 (75.0–86.0)	77.0 (71.0–80.0)	0.022
Median size, mm (IQR)	25.0 (15.0–40.0)	20.0 (4.0–90.0)	25.0 (18.5–40.0)	0.17
Localization, n (%)				
Head and neck	50 (59.5)	43 (81.1)	7 (22.6)	< 0.001
Limb	33 (39.3)	10 (18.9)	23 (74.2)	
Unknown	1 (1.2)	0 (0.0)	1 (3.2)	
Blood disease, n (%)	11 (13.1)	8 (15.1)	3 (9.6)	
TNM, n (%)	31 (36.9)	23 (43.3)	8 (25.8)	0.097
T1	36 (42.9)	19 (35.8)	17 (54.8)	
T2	14 (16.6)	11 (20.8)	3 (9.7)	
T3	3 (3.6)	0	3 (9.7)	
Unknown				
Median radiation dose on the lesion*, Gy, (IQR)	55.0 (50.0–62.0)	60.0 (50.0–65.0)	50.0 (50.0–54.0)	0.003
Median radiation dose on the draining lymph nodes, Gy, (IQR)	50.0 (40.0–50.0)	50.0 (33.0–50.0)	50.0 (50.0–55.0)	0.21

^*^Cumulative total dose

*p* values are obtained using Chi-square tests for categorical characteristics and the Mann–Whitney U test for continuous characteristics

### Treatment characteristics

Most patients received conventional external beam radiotherapy (EBRT) (daily treatment of 2 Gy, 5 fractions a week). Three patients out of 53 in group A (5.6%) were treated with hypofractionated RT (decrease in the number of fractions, but dose per session > 2.2 Gy, median doses of 45 Gy (EQD2 = 48.75 Gy). Median EBRT doses of 60.0 Gy (IQR 50–65) were delivered on the primary tumor with large irradiation fields. Seven patients in group A (13.2%) received 45 Gy or less because of bad tolerance or treatment impact on general well-being. In group B, patients had surgery with margins > 2 cm (maximum 3 cm), followed by adjuvant RT with a median dose of 50 Gy (IQR 50–54 Gy). Four patients in group B (but none in group A) had a negative SLNB.

We performed prophylactic irradiation of the draining lymph nodes and of the area between the primary tumor site and the draining lymph nodes in 64 patients (76.2%). This included 40 out of the 53 patients in group A (75.5%) and 24 out of the 31 patients in group B (77.4%). The median dose was 50 Gy (IQR 50–53) in group A and 50 Gy (IQR 50–55) in group B. The treatment characteristics are presented in Table 1*.*

### Outcomes

**Fig. 2:**
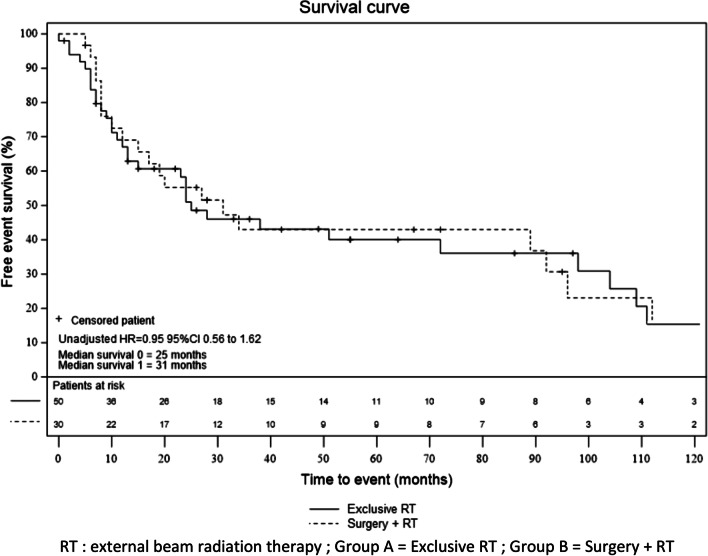
Disease free survival (all relapses included) according to treatment group. RT: external beam radiation therapy; Group A = Exclusive RT; Group B = Surgery + RT. There was no statistical difference, in the unadjusted (HR, 0.95, 95% CI 0.55–1.62) and adjusted model (HR = 1.03 CI 95% 0.50–2.13), between the disease free survival rates in the two groups using Cox regression models. The rates of overall survival were estimated using the Kaplan–Meier method

The median follow-up was 64 months (CI 95% 38–148 months) for group A and 95 months (CI 95% 42–244 months) for group B (*p* = *0.26*). At the end of the follow-up, 57 out of the 82 patients (69.5%) presented a relapse or died (while 27 patients presented local or distant relapse). Of the 57 patients who presented a relapse or died, 34 were in group A (66.6%) and 23 in group B (74.2%) in group B. As shown in Fig. 2, there was no significant difference between the two groups, with a 5-year DFS of 40.0% (CI 95% 25.3–54.4) in group A and 43.0% (CI 95% 24.3–60.4) in group B. Similar results were found in the multivariate analysis adjusted for prespecified confounders, with an adjusted HR of 1.03 (CI 95% 0.50–2.13). Figure 2 presents DFS for each group.

**Fig. 3:**
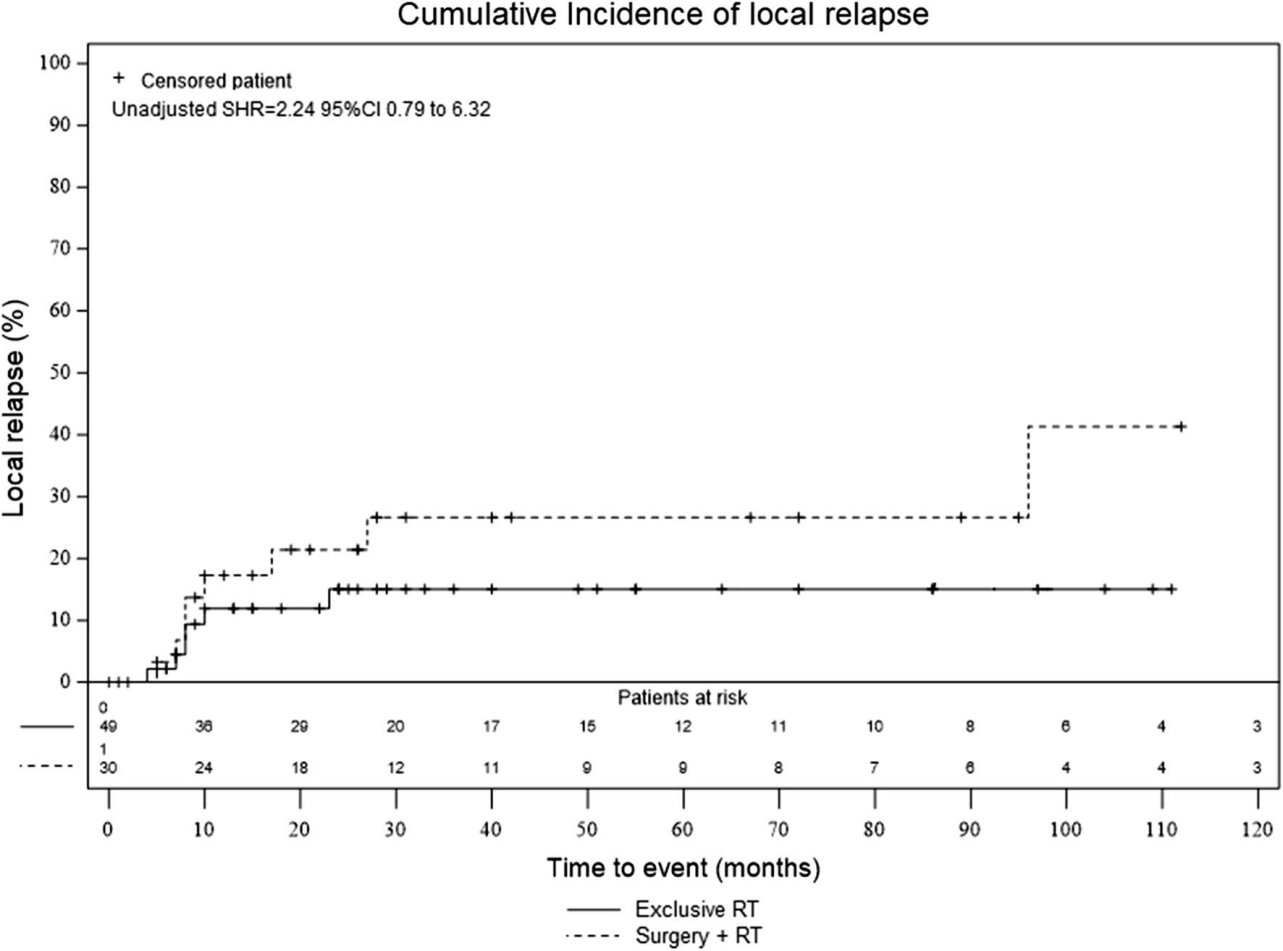
Cumulative incidence of local relapse according to treatment group. There was no statistical difference, in the unadjusted (sHR, 2.24, 95% CI 0.79–6.32) and adjusted model (sHR = 1.47 CI 95% 0.31–7.07), between the local relapse rates in the two groups using Fine-Gray models models. The rates of overall survival were estimated using the approach of Kalbfleisch and Prentice

Among the 27 patients who relapsed, 15 experienced local relapse (17.9%). Those included seven patients in group A (13.7%) and eight patients in group B (25.8%). The 5-year cumulative incidence of local relapse was 17.5% (CI 95% 9.8–27.0) in the entire cohort, 13.0% (CI 95% 5.2–24.5) in group A and 8.3% (CI 95% 10.6–41.7) in group B (adjusted sHR = 1.47, CI 95% 0.31–7.07) (Fig. 3)*.*

Twelve patients (14.2%) presented nodal relapse. Those included eight patients in group A (15.7%) and four patients in group B (12.9%). Among these 12 patients, four haven’t been treated by irradiation on draining lymph nodes. The 5-year cumulative incidence of nodal relapse was 16.0% (95% CI 8.7–25.2) in the entire cohort, 16.7% (95% CI 7.7–28.4) in group A and 14.9% (95% CI 4.5–31.3) in group B [adjusted sHR = 0.72 (0.15–3.44)] (Additional file 1: Supplemental figure 1).

Eighteen patients (21.4%) presented metastatic recurrences. Those included six patients in group A (11.3%) and 12 patients in group B (38.9%). The 5-year cumulative incidence of metastatic relapse was 21.6 (CI 95% 12.6–32.1) in the entire cohort, 11.1% (CI 95% 4.0–22.4) in group A and 37.6% (CI 95% 19.2–56.0) in group B (adjusted sHR = 2.72, CI 95% 0.48–15.24) (Additional file 1: Supplemental figure 2)*.*

**Fig. 4:**
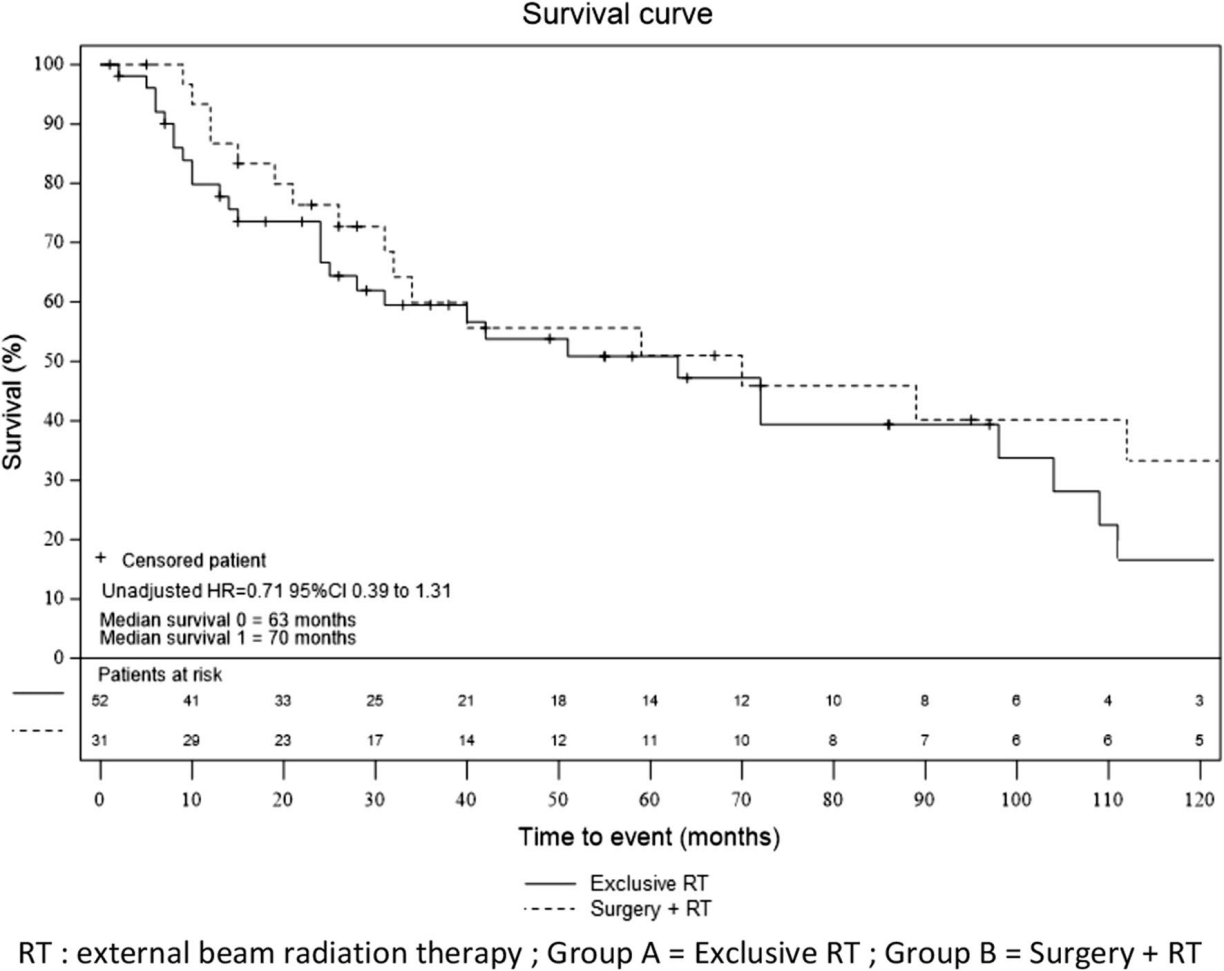
Overall survival according to treatment group. RT: external beam radiation therapy; Group A = Exclusive RT; Group B = Surgery + RT. There was no statistical difference, in the unadjusted (HR, 0.71, 95% CI 0.39–1.30) and adjusted model (HR = 1.05 CI 95% 0.42–2.61), between the overall survival rates in the two groups using Cox regression models. The rates of overall survival were estimated using the Kaplan–Meier method

Forty-eight patients (57.1%) died during the follow-up: 30 in group A (56.7%) and 18 in group B (58.1%). The 5-year OS was 50.7% (CI 95% 38.2–62.0), with 50.8% in group A (CI 95% 34.9–64.7) and 51.0% in group B (CI 95% 30.4–68.4). There was no statistical difference between the overall survival rates in the two groups (adjusted HR = 1.05 CI 95% 0.42–2.61). OS according to the group is shown in Fig. 4*.* 15 of those deaths [5 patients in group A (16.7%) and 9 patients in group B (50.0%)] were related to the MCC. The 5-year specific survival rate was 17.6% (CI 95% 9.8–27.2): 10.6% (CI 95% 3.8–21.4) in group A and 28.9% (CI 95% 13.2–46.7) in group B. There was no statistical difference, in the adjusted model, between the specific survival rates in the two groups (sHR = 7.16 CI 95% 0.77–66.18) (Additional file 1: Supplemental figure 3).

### Irradiation dose

In group A, seven out of 53 patients (13.2%) received an irradiation dose less than or equal to 45 Gy due to bad general conditions or to the occurrence of radiation-induced adverse events. After a median follow-up of 24 months, only one patient had a local relapse at 10 months of diagnosis and was treated successfully by surgery. Another patient had an early metastatic progression 3 months after the diagnosis.

### Radiotherapy dose hypofractionation

In group A, three patients (5.6%) were treated by hypofractionated RT in order to reduce the number of sessions and limit travels for their well-being. None of them had a local relapse. One of them had a nodal relapse at 6 months, which was controlled by subsequent RT. This patient died 10 years later from heart disease.

## Discussion

Merkel cell carcinoma is a rare skin cancer, limiting the possibilities of large cohort studies. To our knowledge, we here report the largest case series on exclusive radiotherapy as a treatment strategy for MCC. Our study confirms that exclusive radiotherapy is an interesting alternative to surgery in localized MCC management.

The importance of adjuvant RT is now well known in the management of MCC. However, surgery is not always feasible in clinical routine due to anatomical issues or sequelae, especially in the head and neck region. Besides, old age and invasiveness of the surgical procedure are predictors of mortality in geriatric patients [16]. The risk of anesthesia-related mortality increases significantly in the elderly population [17]. In addition to the per-operative risks, post-operative confusion and post-operative cognitive dysfunction may occur, even after a minor surgery [18–20] Local anesthesia is sometimes not conceivable in view of the complexity of the surgery, or in people with dementia. It remains a painful technique and may increase stress in some patients [21]. The elderly appear to be at a disproportionately increased risk for toxicity owing to the presence of relevant comorbidities and decreased muscle mass [22].

Our population is overall comparable to previously published data on MCC, on median age and median size of tumors [11–13]. As expected, the primitive tumor was frequently found in the cephalic region in our cohorts. This predominance is less underlined in other studies. Nevertheless, the distribution of primary tumor sites was not homogeneous in the two groups, with a clear predominance of cervicocephalic tumors in the RT monotherapy group (Group A). This result can be explained by a greater reluctance of clinicians to operate face-tumors due to risks of unaesthetic scars. Prognosis seems to be worse for head and neck tumors compared to limbs according to previous data [23], but despite a predominance of these lesions in the group A, no difference was found after adjustment on the localization.

Irradiation doses were similar to our data, with median doses to the primary site of 51 Gy (range 20–63) in 2 Gy fractions in study of Veness et al. and 52.1 Gy in study of Harrington et al. The median nodal-site dose was 50 Gy (range 20–64) in the study of Veness, which is slightly lower.

We observed a 13.7%(95% CI 8.0–43.7) local recurrence rate in group A which is comparable to previously published studies [12, 13]. Our results showed a 5-year OS of 50.8% (CI 95% 34.9–64.7) in the RT monotherapy group, which is slightly higher than previously reported^43^. Harrington et al., report a 40.0% 5-year OS, but this study included a large proportion of stage III patients [13]. More patients in group B died from MCC (50%, compared to 16.0% in group A). All group A patients died from another cause (16 of them from organ failure, 2 of strokes, 1 of hematological disease and 1 of another solid cancer), which demonstrates that an overly aggressive approach is not necessary for localized MCC.

The 5-year DFS was 40.0% in our study, which is lower than the study of Harrington (57%) and Veness (46%).

One of the strengths of our study was to choose exclusive RT strategy in patients with a tumor in place at the time of irradiation. Contrary to other studies, there was no resection even for cleanliness or debulking prior to irradiation. This approach was closer to clinical daily routine because of the difficult resection of these tumors. Furthermore, elderly people are characterized by a greater frailty, increasing their vulnerability to poor resolution of homoeostasis after a stress event. An apparently petty act (as minor surgery) could result in disproportionate changes in the health state [24]. Our study suggests that surgery could be avoided in some cases.

Limitations of our study are its monocentric and retrospective nature. In addition, we excluded a significant proportion of patients treated by surgery with insufficient margins.

Indeed, few patients were treated by RT and surgery with margins > 2 cm. Still, the studies carried out in our center lead us to believe that RT is an essential step in the treatment of MCC. We believe that it is imperative not to delay the RT, and for this reason, we have reduced margins and promoted surgery allowing a direct suture, in order to obtain a rapid wound healing and early irradiation.

Four patients in our series had a SLNB, all in group B and with negative SLNB. This technique was not performed for MCC until 2010 and many patients treated by exclusive RT had contraindications to anaesthesia. Despite the risk of undiagnosed micrometastases in Group A patients, no difference was shown between the two groups.

Radiation dose was higher in group A than in group B. In addition, none of the three patients who were treated by hypofractionated RT had a local relapse. Hypofractionated RT requires less hospital visits for patients compared to other techniques. It should nevertheless be reserved for elderly subjects, because it increases the risk of late toxicities. Brachytherapy could also be an interesting option in elderly subjects for lesions of the extremities, with good efficacy, tolerance and aesthetic results [25, 26].

## Conclusions

Our findings suggest that exclusive radiotherapy is a promising treatment for early-stage MCC, even where the patients have not had prior surgery. This approach could be useful in elderly patients with comorbidities, or when surgery would cause significant functional or aesthetic sequelae.

## Supplementary Information

**Additional file 1: Supplemental figure 1**. There was no statistical difference, in the unadjusted (sHR, 0.78, 95% CI 0.24 to 2.51) and adjusted model (sHR = 0.72 CI95% 0.15 to 3.44), between the local relapse rates in the two groups using Fine-Gray models models. The rates of overall survival were estimated using the approach of Kalbfleisch and Prentice.

## Data Availability

All data generated and analyzed during this study are included in this published article (and its supplementary information files).

## Abbreviations

CNIL: Commission nationale Informatique et Libertés
DFS: Disease free survival
IQR: Interquartile range
MCPyV: Merkel cell polyomavirus
MCC: Merkel cell carcinoma
OS: Overall survival
RT: Radiotherapy
SLNB: Sentinel lymph node biopsy
