# Supplementary material for: Outcome of early stage Merkel carcinoma treated by exclusive radiation: a study of 53 patients
Source: Radiat Oncol. 2021 May 14;16:90. doi: 10.1186/s13014-021-01815-4 (PMC8120723; doi:10.1186/s13014-021-01815-4)
Supplement: Supplementary file 1 — Additional file 1: Supplemental figure 1. There was no statistical difference, in the unadjusted (sHR, 0.78, 95% CI 0.24 to 2.51) and adjusted model (sHR = 0.72 CI95% 0.15 to 3.44), between the local relapse rates in the two groups using Fine-Gray models models. The rates of overall survival were estimated using the approach of Kalbfleisch and Prentice. [file 13014_2021_1815_MOESM1_ESM.docx]

Supplemental Figure 1


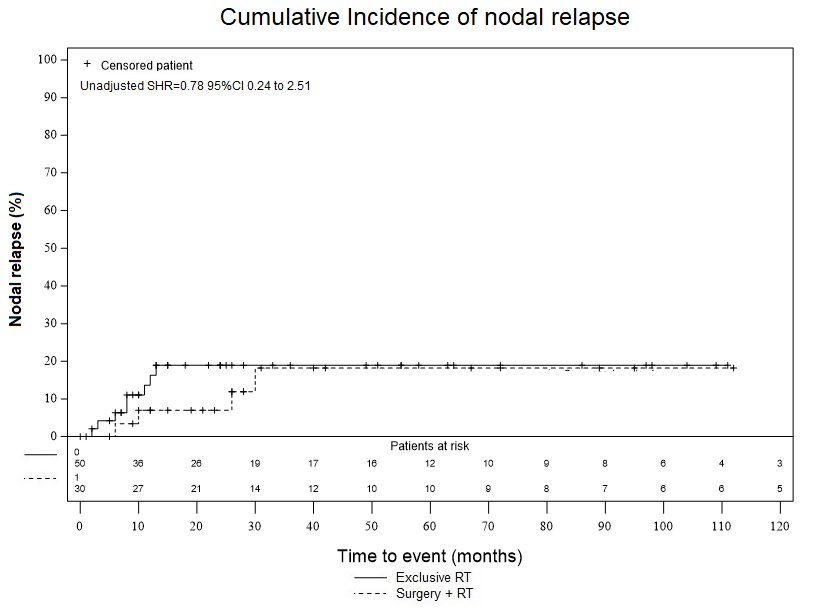


Supplemental figure 1: There was no statistical difference, in the unadjusted (sHR, 0.78, 95%CI 0.24

to 2.51) and adjusted model (sHR=0.72 CI95% 0.15 to 3.44), between the local relapse rates in the

two groups using Fine-Gray models models. The rates of overall survival were

Supplemental Figure 2


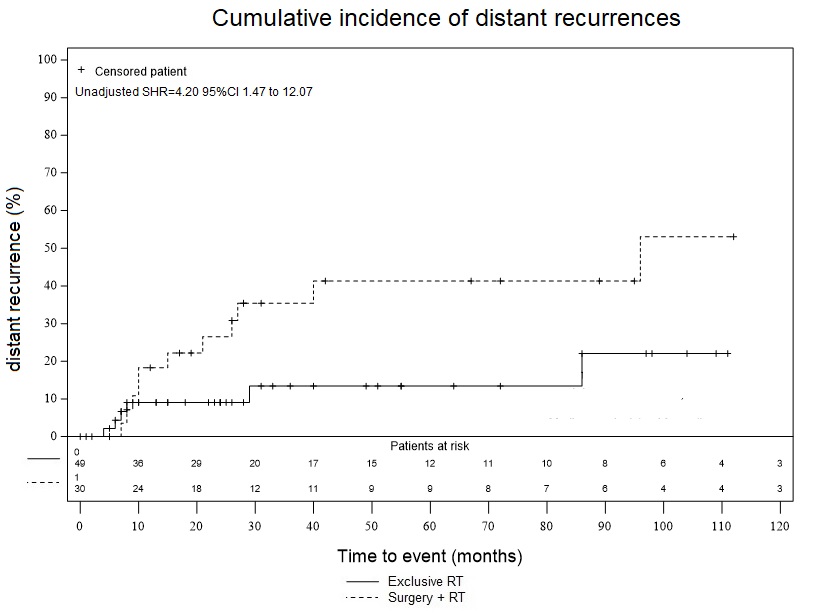


Supplemental Figure 2: There was no statistical difference, in the unadjusted (sHR, 4.20, 95%CI 1.47 to 12.07) and adjusted model (sHR=2.72 CI95% 0.48 to 15.24), between the local relapse rates in the two groups using Fine-Gray models models. The rates of overall survival were estimated using the approach of Kalbfleisch and Prentice.

Supplemental Figure 3


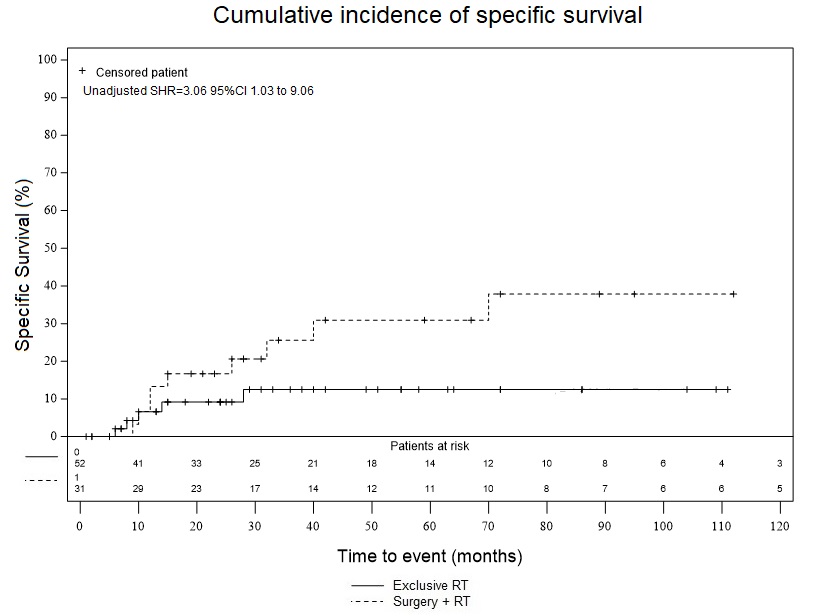


Supplemental figure 3 : There was no statistical difference, in the unadjusted (sHR, 3.06, 95%CI 1.03

to 9.06) and adjusted model (sHR=7.16 CI95% 0.77 to 66.18), between the local relapse rates in the two

groups using Fine-Gray models models. The rates of overall survival were estimated using the

approach of Kalbfleisch and Prentice.
